# Spatial Analysis of Drug-Resistant Tuberculosis in Colombia (2020–2023): Departmental Rates, Clusters, and Associated Factors

**DOI:** 10.3390/tropicalmed10120351

**Published:** 2025-12-15

**Authors:** Brayan Patiño-Palma, Sandra Chacon-Bambague, Farlhyn Bermudez-Moreno, Carmencita Peña-Briceño, Juan Bustos-Carvajal, Florencio Arias-Coronel

**Affiliations:** 1 Faculty of Health and Sports Sciences, Fundación Universitaria del Área Andina, Pereira 660004, Colombia; bpatino3@areandina.edu.co (B.P.-P.); schacon3@areandina.edu.co (S.C.-B.); fbermudez@areandina.edu.co (F.B.-M.); cpena14@areandina.edu.co (C.P.-B.); 2 Faculty of Health, Universidad Autónoma de Manizales, Manizales 170004, Colombia; 3 Universidad de Pamplona, Pamplona 540004, Colombia; juan.bustos2@unipamplona.edu.co; 4 Faculty of Health, Universidad Santiago de Cali, Cali 760035, Colombia

**Keywords:** drug-resistant tuberculosis, spatial analysis, Colombia, epidemiology, public health, clusters, risk factors, infectious diseases, surveillance

## Abstract

Background: Drug-resistant tuberculosis (DR-TB) constitutes a serious threat to global public health due to the increase in strains resistant to multiple drugs, especially isoniazid and rifampicin. This resistance increases mortality, estimated at 25.6% globally, and complicates treatments due to its high toxicity and cost. Materials and Methods: A quantitative ecological study was carried out with data on drug-resistant tuberculosis reported in Sivigila in the years (2020–2023) SIVIGILA database. 1694 cases were analyzed, considering sociodemographic variables such as age, sex, nationality and prioritized population groups. Departmental rates per 100,000 inhabitants were calculated with DANE projection, from these choropleth maps were developed. Applying a Kulldorff spatial scan under a Poisson model using the SMERC package of R (version 4.5.1), with windows centered on each department and Monte Carlo simulation contrast to identify high-risk clusters (RR > 1). Results: (DR-TB) Predominantly in men aged 30–44 years, with a progressive increase until 2023 (IRR = 2.11). Three high-risk clusters were detected in the southwest and center of the country. Discussion: Drug-resistant tuberculosis in Colombia showed a sustained increase in the years of study, with a cumulative increase of 110% compared to 2020, associated with economically active people more exposed due to occupational and social factors. The greatest burden was observed in the general population. Cases also increased in groups with social and health vulnerability conditions. Conclusions: The departments of Risaralda, Meta, and Valle del Cauca presented the highest drug resistance rates in Colombia.

## 1. Introduction

Tuberculosis is a severe infectious disease caused by Mycobacterium tuberculosis, primarily affecting the lungs but capable of involving other organs such as lymph nodes, kidneys, the central nervous system, and the spine, in which case it presents as extrapulmonary tuberculosis [1]. The disease typically begins with progressive symptoms—persistent cough, fever, night sweats, weight loss, lymphadenopathy, and general weakness—and, in advanced stages, may manifest with chest pain and hemoptysis [2].

Drug-resistant tuberculosis (DR-TB) refers to disease caused by Mycobacterium tuberculosis complex strains that exhibit resistance to one or more antituberculosis medicines, according to the World Health Organization [3]. This includes resistance to isoniazid and rifampicin, the main first-line agents used in standard treatment regimens [3].

Globally, DR-TB represents a growing threat to public health, particularly in high-burden countries [4]. The emergence of new resistant variants has further complicated treatment and contributed to rising mortality [5]. According to the meta-analysis conducted by Alemu et al. [4] worldwide mortality attributable to DR-TB average mortality rate of 25.6% (95% CI: 20.9–30.3) during treatment, this is equivalent to 3.75 deaths per 10,000 people infected, which shows that approximately one in four patients with resistant tuberculosis dies before completing therapy. If we analyze these results disaggregated by the type of resistance, a marked difference is observed: MDR-TB had a mortality of 20.2%, while extensively resistant tuberculosis (XDR-TB) reached 43.5%, which represents almost double the risk of death, reflecting a clear relationship between the magnitude of bacterial resistance and lethality.

Aligned with the Sustainable Development Goals, the World Health Organization (WHO) has set the elimination of tuberculosis by 2030 as a global priority. However, in 2023 an estimated 10.8 million new TB cases occurred worldwide, and only two out of five individuals diagnosed with DR-TB were able to access treatment [6].

From a global epidemiological perspective, tuberculosis remains a structural marker of health inequality. The disease is disproportionately concentrated in low- and lower-middle-income countries, where incidence and mortality rates are several times higher than in high-income regions. This distribution reflects profound social determinants—including overcrowding, low educational attainment, fragile health systems, poor living conditions, and cumulative exposure to environmental and social risks—rather than a purely biological distribution of the pathogen [7,8].

In this context, Chen et al. [9], report that MDR–TB and XDR–TB represented, in 2021, a critical global health burden, with 443,680 and 24,036 incident cases, respectively, mostly in developing regions. These resistant forms drive disproportionate mortality and underscore structural failures in early diagnosis and health system resilience.

Regionally, Eastern Europe, Central Asia, and parts of Africa exhibit the highest concentration of DR-TB, associated with factors such as HIV co-infection, socioeconomic precariousness, incomplete previous TB treatment, diabetes mellitus, and harmful habits including smoking and alcohol consumption. The persistence of these factors complicates timely recovery even when effective therapeutic options exist [10].

In Latin America, Mexico, despite being classified as a low-burden country, has reported resistance in 14.1% of analyzed strains, with notable discrepancies between phenotypic and genotypic profiles, suggesting non-canonical variants or new mutations [11]. In Ecuador, where TB burden is also relatively low, 10.9% of tested isolates were multidrug-resistant, some likely introduced from neighboring high-prevalence regions such as Venezuela, emphasizing the need for strengthened molecular surveillance [12].

In Colombia, the 2022 incidence of DR-TB was 0.67 per 100,000 inhabitants, with a slight increase from the previous year. Although 60% of DR-TB cases were reported as cured, significant challenges persist in clinical management. The COVID-19 pandemic further disrupted TB control, causing delays in diagnosis, interruptions in follow-up, and reduced timely detection, thereby worsening the national DR-TB situation [13].

Given this scenario, it is essential to understand the territorial distribution of DR-TB within the country. This study aims to analyze the geographic distribution and spatial patterns of DR-TB in Colombia between 2020 and 2023 through spatial analysis techniques oriented toward identifying territorial clusters with high epidemiological burden. This approach supports decision-making, optimizes resource allocation, and strengthens targeted control interventions; particularly in areas with high social and health vulnerability.

## 2. Materials and Methods

An ecological study with a quantitative approach was conducted using all reported cases of tuberculosis in Colombia between 2020 and 2023. The information was obtained from the Public Health Surveillance System (SIVIGILA) of the National Institute of Health (INS) and downloaded in June 2025. For the analysis, only the records corresponding to the “Drug-resistant tuberculosis” event (code 825) were selected, from which a specific analytical sub-database was constructed.

Sociodemographic variables included age, sex, nationality, and belonging to prioritized population groups (disability, displacement, migration, deprivation of liberty, pregnancy, indigenous population, among others). Ethnicity-related variables were excluded because of conceptual overlap with the prioritized population categories. Likewise, the variables “case type” and “area” were excluded due to their limited analytic relevance within this cohort.

The age variable was initially treated as continuous and subsequently categorized into six intervals (0–14, 15–29, 30–44, 45–59, 60–74, and ≥75 years). This structure follows the categorization used in national INS tuberculosis surveillance reports and WHO epidemiological classifications, ensuring comparability across official monitoring systems. The intervals also correspond to meaningful demographic groups that differ in exposure patterns and clinical presentation.

For the descriptive analysis, a general table was constructed including absolute frequencies, percentages, and 95% confidence intervals. For the spatial component, the official national shapefile of Colombian municipalities was obtained from the GEOPORTAL of the National Administrative Department of Statistics (DANE). The data were aggregated at the departmental level. To ensure consistent denominators, a complete department–year grid (33 departments × 4 years) was constructed and merged with the notification database. Department–year cells without DR-TB reports were assigned a value of zero. Annual departmental rates were calculated using the official DANE population projections as denominators:

$$Rate\;per\;100,000=\;\frac{DR-TB\;notifications}{Population}\times 100,000$$

The national annual rates were then calculated by adding the cases and population from all 33 departments, resulting in a true population-based national indicator that is consistent across figures and tables. Choropleth maps based on these values were created using a continuous linear color scale. Cumulative departmental rates for 2020–2023 were also calculated by adding cases and populations over four years and computing the corresponding Poisson exact confidence intervals.

To evaluate the association between year of notification and prioritized population groups with the number of DR-TB cases, three Poisson regression models were compared: Model A (Cases~YEAR), Model B (Cases~YEAR + GROUP), and Model C, which incorporated the interaction term between both factors (Cases~YEAR × GROUP). Model selection was based on Akaike (AIC) and Bayesian (BIC) information criteria, as well as likelihood ratio tests (LRT) for nested models. In this analysis, IRRs describe relative changes in the number of notifications between categories (not risk per capita), using 2020 and the size of the general population as a reference.

Model B demonstrated a drastically superior fit compared with Model A (AIC: 184 vs. 6696; BIC: 212 vs. 6704; McFadden R^2^ = 0.977 vs. 0.0195), and the LRT confirmed a highly significant improvement in explanatory power (Deviance = 6534, *p* < 0.001). Although Model C produced a marginally higher McFadden R^2^ (0.983), it yielded worse information criteria (AIC = 213; BIC = 302) and exhibited severe numerical instability, reflected in an infinite dispersion parameter (φ = Inf). Moreover, the LRT comparing Model C with Model B showed no statistically significant improvement (Deviance = 37.5, *p* = 0.27). Given the absence of meaningful gains in model performance, the instability associated with multiple interaction terms in low-frequency categories, and the marked penalization for complexity reflected in the BIC, Model B was selected as the final specification.

Finally, a Kulldorff-type spatial scan analysis was conducted under a Poisson model using the smerc package in R. Circular scanning windows were centered on each department, and statistical significance was assessed through Monte Carlo simulation. To avoid excessively large or non-informative clusters, the maximum cluster size was restricted to 50% of the population at risk, and only non-overlapping clusters with *p* < 0.05 were reported.

## 3. Results

A total of 1694 cases of DR-TB were analyzed. The highest frequencies were observed in the age groups of 30–44 years (26.1%), 15–29 years (24.5%), and 45–59 years (21.8%). The age group of 60–74 years also represented a substantial proportion of cases (17.9%), whereas age extremes contributed markedly fewer notifications: 8.3% in adults aged ≥ 75 years and 1.5% in children under 15 years. Most cases occurred in males (66.9%). Regarding nationality, 94.9% of patients were Colombian and 5.1% were Venezuelan.

In relation to population groups, 88.3% of cases were categorized as belonging to the general population, which is a residual group made up of people who do not fit the requirements for any particular prioritized category. Indigenous people (2.8%), migrants (4.3%), and those deprived of liberty (5.5%) showed lower but noteworthy frequencies. Less than 1% of the total was made up of other groups, such as individuals with mental disorders, pregnant women, victims of violence, people with disabilities, and displaced people. Table 1 provides complete descriptive data, including percentages, frequencies, and 95% confidence intervals.

**Figure 1:**
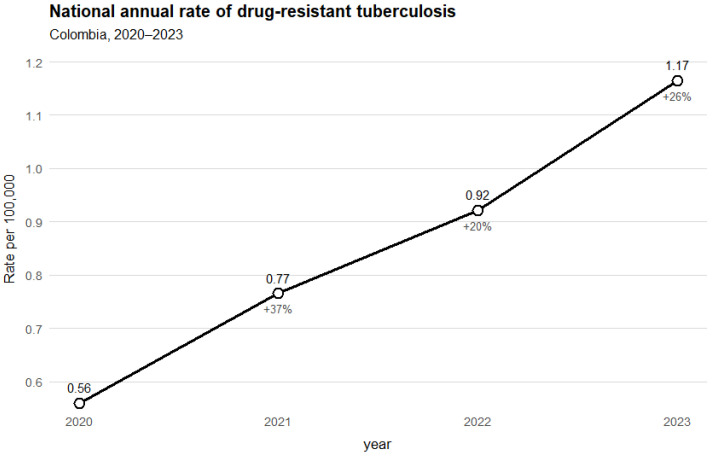
National annual rate of DR-TB, Colombia, 2020–2023. Case source: SIVIGILA (download: June 2025). Population denominators: DANE (departmental projection).

The temporal distribution of drug-resistant TB is presented in Figure 1. The national rate increased steadily from 0.56 cases per 100,000 inhabitants in 2020 to 1.17 in 2023, with year-on-year variations of +37%, +20%, and +26%, respectively.

**Figure 2:**
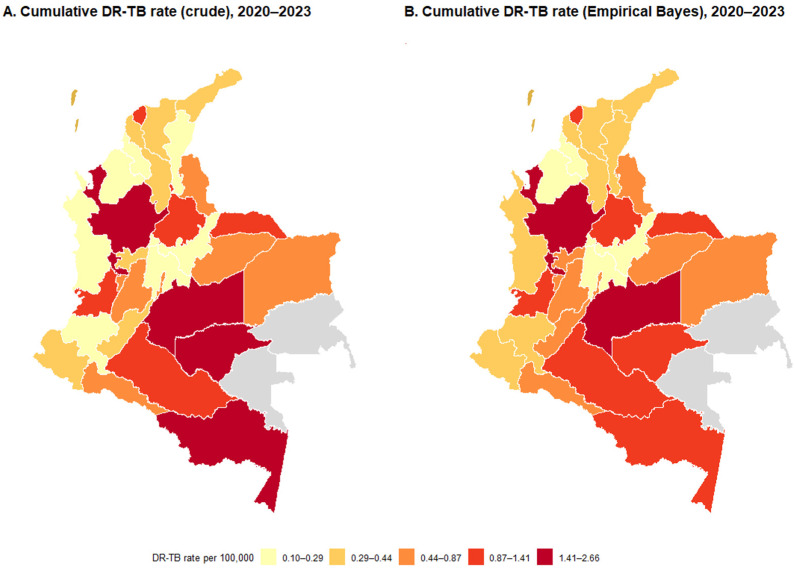
Cumulative rate of DR-TB by department, Colombia, 2020–2023: (**A**) crude rate and (**B**) neighborhood-stabilized rate (empirical Bayesian method), per 100,000 inhabitants. Case source: SIVIGILA (download: June 2025). Population denominators: DANE (departmental projection).

After the general characterization of cases, the spatial distribution of the cumulative DR-TB rate per 100,000 inhabitants for the period 2020–2023 is presented in Figure 2. The figure includes two panels for comparison: the crude cumulative rate (left) and the empirical Bayesian-stabilized rate (right). A magnified inset shows the San Andrés Archipelago. The scales and cuts are identical for easy comparison.

In addition to Figure 2, Table S1 presents the annual DR-TB rates for all Colombian departments from 2020 to 2023, together with their corresponding 95% confidence intervals. The national annual values increased from 0.56 cases per 100,000 inhabitants in 2020 to 1.17 per 100,000 inhabitants in 2023. Considering the full study period, the cumulative national rate reached 0.86 cases per 100,000 inhabitants. Figure S1 displays the annual departmental maps using consistent scales and breakpoints to facilitate visual comparison across years. Areas shown in gray correspond to territories without available data after the harmonization process.

The cumulative departmental rates reported in Table S1 show a wide range of values across the country. The highest cumulative rates correspond to Risaralda (0.665 per 100,000 inhabitants), Meta (0.452), Valle del Cauca (0.326), Caquetá (0.298), and Guaviare (0.268). Departments such as Santander, Tolima, Arauca, and Quindío show intermediate cumulative rates, whereas Córdoba, Cundinamarca, La Guajira, César, Boyacá, and Sucre report lower cumulative values. Table S1 provides the exact annual rates and their confidence intervals for each department.

All prioritized population groups defined by the national surveillance system recorded at least one DR-TB notification between 2020 and 2023. Most notifications correspond to individuals classified within the general population. Among the prioritized groups, the highest number of notifications is observed in persons deprived of liberty, followed by migrants and indigenous populations. Other groups, such as individuals with mental disorders, pregnant women, persons with disabilities, victims of violence, and displaced individuals; show lower annual counts throughout the four-year period.

To evaluate the association between the different variables of interest with the number of cases of drug-resistant tuberculosis, a Poisson regression with main effects was adjusted. The results are expressed as incidence rate ratios (IRR) with 95% CI and *p*-values, taking 2020 (year) and general population (group) as references. Table 2 presents the estimated IRRs for each year category and prioritized groups, along with their confidence intervals and statistical significance.

The selected model showed a progressive and statistically significant increase in DR-TB notifications compared with 2020. For 2021, the IRR was 1.28 (+28%); for 2022, 1.71 (+71%); and for 2023, 2.11 (+111%), indicating a sustained year-on-year rise in reported cases. This pattern is consistent with the upward trend observed in the national annual notification rate.

In terms of prioritized groups, all categories had IRRs significantly below 1 (e.g., migrants: ≈0.05; persons deprived of liberty: ≈0.06; indigenous population: ≈0.02), meaning that their contribution to notifications during the study years was lower than that of the general population. As mentioned earlier, these IRRs should be interpreted with caution because the model does not account for group-specific denominators, which means that variations in notification counts rather than population-adjusted risks are reflected.

The overall performance of the model was adequate, with low information criteria (AIC/BIC) relative to its complexity, a high McFadden pseudo-R^2^, and only mild overdispersion (φ = 1.44), which does not compromise the interpretation of the main effects. In summary, the model supports a sustained temporal increase in DR-TB notifications and highlights structural differences between groups in their relative contribution to reported cases.

**Figure 3:**
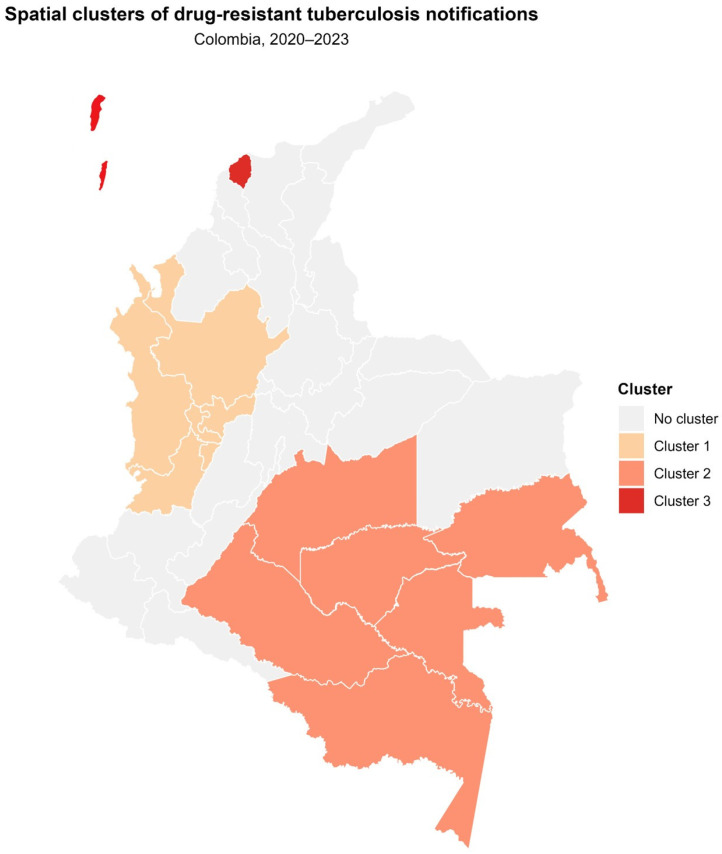
Spatial clusters of DR-TB with elevated relative risk in Colombia, 2020–2023. RR: observed/expected ratio estimated using Kulldorff’s spatial scan (Poisson model). Values > 1 indicate an excess of cases; the *p*-value is obtained through Monte Carlo simulation.

A Kulldorff-type spatial scan using Poisson’s model was applied to detect areas with significant aggregation of DR-TB cases. The departments identified as statistically significant high-risk clusters during the 2020–2023 analysis periods are shown in Figure 3.

The spatial scan analysis identified three statistically significant clusters of drug-resistant tuberculosis notifications for the 2020–2023 period (*p* < 0.05) (Figure 3).

Cluster 1 comprised six departments: Antioquia, Caldas, Chocó, Quindío, Risaralda, and Valle del Cauca, and accounted for 826 notifications. This cluster showed a relative risk (RR) of 1.44 compared with the national distribution. Cluster 2 encompassed Caquetá, Meta, Amazonas, Guaviare, Vaupés, and Guainía, forming a contiguous area in the south-eastern region of the country. A total of 106 notifications were recorded within this cluster, which presented the highest relative risk among the detected clusters (RR = 1.66). Cluster 3 included Atlántico and San Andrés y Providencia, with 156 notifications during the study period and a relative risk of 1.41.

The remaining 19 departments were not part of statistically significant clusters and collectively contributed 606 notifications, corresponding to a cumulative relative risk of 0.49.

## 4. Discussion

In Colombia, drug-resistant tuberculosis (DR-TB) notifications increased steadily from 2020 to 2023. The Poisson regression model indicated a year-to-year rise in reported cases relative to 2020, with increments of 28% in 2021, 71% in 2022, and 110% in 2023. The spatial scan analysis identified three statistically significant clusters (*p* < 0.05): (i) a central–western group comprising Antioquia, Caldas, Chocó, Quindío, Risaralda, and Valle del Cauca; (ii) a south-eastern cluster including Caquetá, Meta, Amazonas, Guaviare, Vaupés, and Guainía; and (iii) a Caribbean cluster formed by Atlántico and San Andrés y Providencia. These clusters corresponded to areas with elevated relative risk during the study period.

The sociodemographic distribution showed the highest concentration of notifications among adults aged 30–44 years, followed by individuals aged 15–29 and 45–59 years. Older adults reported fewer cases overall. Men accounted for 66.9% of notifications, and most cases originated from the general population (88.3%), while the deprived-of-liberty population, migrants, and indigenous groups contributed smaller absolute counts.

The predominance of cases among young and middle-aged adults aligns with reports indicating higher DR-TB burden among populations of productive age, particularly in conditions of social vulnerability [14]. Although older adults contributed fewer notifications in this study, international evidence shows that they often experience more severe disease and greater lethality [15].

The marked gender imbalance observed, where men constitute the majority of DR-TB, cases is consistent with studies demonstrating gendered differences in biological susceptibility, exposure patterns, and social determinants [16]. Peer et al. further demonstrate that the probability of DR-TB is higher in men across the life course, shaped by hormonal transitions, occupational exposures, and gender norms [17].

The contribution of Venezuelan migrants reflects regional trends documented by PAHO, which describes increased TB risk in displaced populations due to precarious housing, food insecurity, and reduced access to health services [18]. Systematic reviews similarly highlight elevated TB susceptibility among refugees and migrants [19], and European evidence suggests that the odds of MDR/RR-TB may be up to threefold higher in-migrant populations compared with non-migrants [20].

The increasing notifications observed among deprived-of-liberty individuals and indigenous populations are coherent with ecological studies from Brazil, where overcrowding, low schooling, and social marginalization contribute to sustained transmission of DR-TB within correctional facilities [21]. Global analyses have also emphasized the disproportionate TB burden among Indigenous peoples, attributable to historical inequities, environmental disadvantage, and both geographic and cultural barriers to care [22,23].

The sharp post-pandemic rise in DR-TB notifications between 2021 and 2023 mirrors global patterns attributed to the disruption of TB control programs during COVID-19, followed by a rebound in diagnostic activity [24]. The World Health Organization has reported the highest number of TB notifications in more than three decades following the pandemic [25]. Similarly, in South Korea, overall TB incidence declined in recent years, yet the proportion of DR-TB cases within total TB notifications increased [26], reflecting complex post-COVID epidemiological dynamics.

The Poisson regression model showed significant temporal increases and improved model fit after incorporating prioritized population groups. Although IRR values for these groups were <1, this should not be interpreted as lower risk. Instead, these values reflect small population denominators in groups such as deprived-of-liberty individuals, pregnant women, migrants, and Indigenous peoples. Because the model compares absolute counts and not per capita rates, lower IRRs reflect population size rather than epidemiological risk. This does not exclude the possibility of higher proportional burden or underdiagnosis among vulnerable groups, as underscored by Litvinjenko et al. [27].

The three spatial clusters identified demonstrate that DR-TB transmission in Colombia is geographically focalized, shaped by dynamics related to urbanization, socioeconomic disadvantage, internal migration, environmental conditions, and structural barriers to care. Evidence from South Africa shows that cross-municipality mobility, especially between urban and periurban districts, facilitates TB and DR-TB spread [28], while hotspots documented in other high-burden settings exhibit similar territorial concentration [29]. The fact that some departments, such as Risaralda, Meta, and Valle del Cauca, exceed the national adjusted incidence rate reinforces the need for geographically differentiated interventions.

This study has methodological strengths, including the use of a national four-year surveillance dataset, systematic comparison of Poisson model specifications, and advanced spatial analyses through Kulldorff scanning to detect high-risk clusters. These approaches provide robust insights into the spatiotemporal dynamics of DR-TB in Colombia.

However, limitations must be acknowledged. The surveillance data did not include clinical variables (e.g., treatment history, comorbidities), socioeconomic indicators, or group-specific denominators, restricting causal inference and requiring cautious interpretation of IRR measures. As with all routine surveillance systems, underreporting and misclassification are possible. Future research should integrate individual-level clinical, social, and environmental data and improve completeness of reporting to refine the understanding of DR-TB transmission patterns.

## 5. Conclusions

The results of this study highlight clear geographic heterogeneity in DR-TB notifications in Colombia. Three statistically significant clusters were identified through spatial scanning: a central–western group (Antioquia, Caldas, Chocó, Quindío, Risaralda, and Valle del Cauca), a south-eastern cluster spanning the Amazon and Orinoquía regions (Caquetá, Meta, Amazonas, Guaviare, Vaupés, and Guainía), and a Caribbean cluster consisting of Atlántico and San Andrés y Providencia. These areas presented elevated relative risks compared with the national distribution and warrant strengthened and locally adapted public health actions.

Further research would benefit from mixed-method approaches capable of capturing structural and contextual barriers affecting individuals affected by DR-TB. Future analyses at finer geographic scales may help identify more specific focal patterns, while integrating clinical and sociodemographic information at the individual level could improve understanding of the determinants of resistant TB in Colombia. Evaluating the performance of current DR-TB control strategies within diverse territorial contexts is also recommended.

## Figures and Tables

**Table 1:** Sociodemographic distribution and membership in prioritized population groups among DR-TB cases in Colombia, 2020–2023 (*n* = 1694).

Variable	Category	Frequency	Percentage	95% CI
Age group	0–14	25	1.5	0.9–2.1
	15–29	415	24.5	22.5–26.5
	30–44	442	26.1	24.0–28.2
	45–59	369	21.8	19.8–23.7
	60–74	303	17.9	16.1–19.7
	75+	140	8.3	7.0–9.6
Sex	Female	560	33.1	30.8–35.3
	Male	1134	66.9	64.7–69.2
Nationality	Colombia	1607	94.9	93.8–95.9
	Venezuela	87	5.1	4.1–6.2
Population with disability	Yes	11	0.6	0.3–1.0
	No	1683	99.4	99.0–99.7
Displaced population	Yes	10	0.6	0.2–1.0
	No	1684	99.4	99.0–99.8
Migrant population	Yes	73	4.3	3.3–5.3
	No	1621	95.7	94.7–96.7
Population deprived of liberty	Yes	93	5.5	4.4–6.6
	No	1601	94.5	93.4–95.6
Pregnant women	Yes	5	0.3	0.0–0.6
	No	1689	99.7	99.4–100.0
Indigenous population	Yes	48	2.8	2.0–3.6
	No	1646	97.2	96.4–98.0
Population with mental disorder	Yes	1	0.1	0.0–0.2
	No	1693	99.9	99.8–100.0
Victims of violence	Yes	1	0.1	0.0–0.2
	No	1693	99.9	99.8–100.0
General population	Yes	1496	88.3	86.8–89.8
	No	198	11.7	10.2–13.2

Values correspond to absolute frequencies, proportions (%), and 95% confidence intervals calculated using the Wald method. Membership in prioritized groups corresponds to the categories defined by the SIVIGILA surveillance system. The category “General population” refers to cases that do not belong to any prioritized group.

**Table 2:** Estimated coefficients of the Poisson regression model for the number of DR-TB cases by year of notification and prioritized population group (Colombia, 2020–2023).

Term (References: Year 2020; General Population)	IRR	95% CI Lower	95% CI Upper	*p*
Year 2021	1.28	1.10	1.50	0.002
Year 2022	1.71	1.48	1.98	<0.001
Year 2023	2.11	1.83	2.43	<0.001
Group: Population migrant	0.05	0.04	0.06	<0.001
Group: Population deprived of liberty	0.06	0.05	0.08	<0.001
Group: Population indigenous	0.02	0.02	0.02	<0.001

Poisson regression (Model B: Year + Group). Colombia, 2020–2023. Effect measure: IRR (Incidence Rate Ratio) with 95% CI. Model fit indicators: AIC = 178.0, BIC = 197.0, pseudo-R^2^ (McFadden) = 0.974, dispersion (φ) = 1.44. Two-tailed *p*-values; significance highlighted at *p* < 0.05. Abbreviations: IRR = Incidence Rate Ratio; 95% CI = 95% Confidence Interval.

## Data Availability

https://portalsivigila.ins.gov.co/, https://www.dane.gov.co/index.php/estadisticas-por-tema/demografia-y-poblacion/proyecciones-de-poblacion, accessed on 15 July 2024.

## Supplementary Materials

The following supporting information can be downloaded at: https://www.mdpi.com/article/10.3390/tropicalmed10120351/s1. Table S1: Annual and Cumulative Rates by Departments; Figure S1: Annual DR-TB Rate by Department (2020–2023).
