# Supplementary figures and images for: Spatial Analysis of Drug-Resistant Tuberculosis in Colombia (2020–2023): Departmental Rates, Clusters, and Associated Factors
Source: Trop Med Infect Dis. 2025 Dec 15;10(12):351. doi: 10.3390/tropicalmed10120351 (PMC12737329; doi:10.3390/tropicalmed10120351)

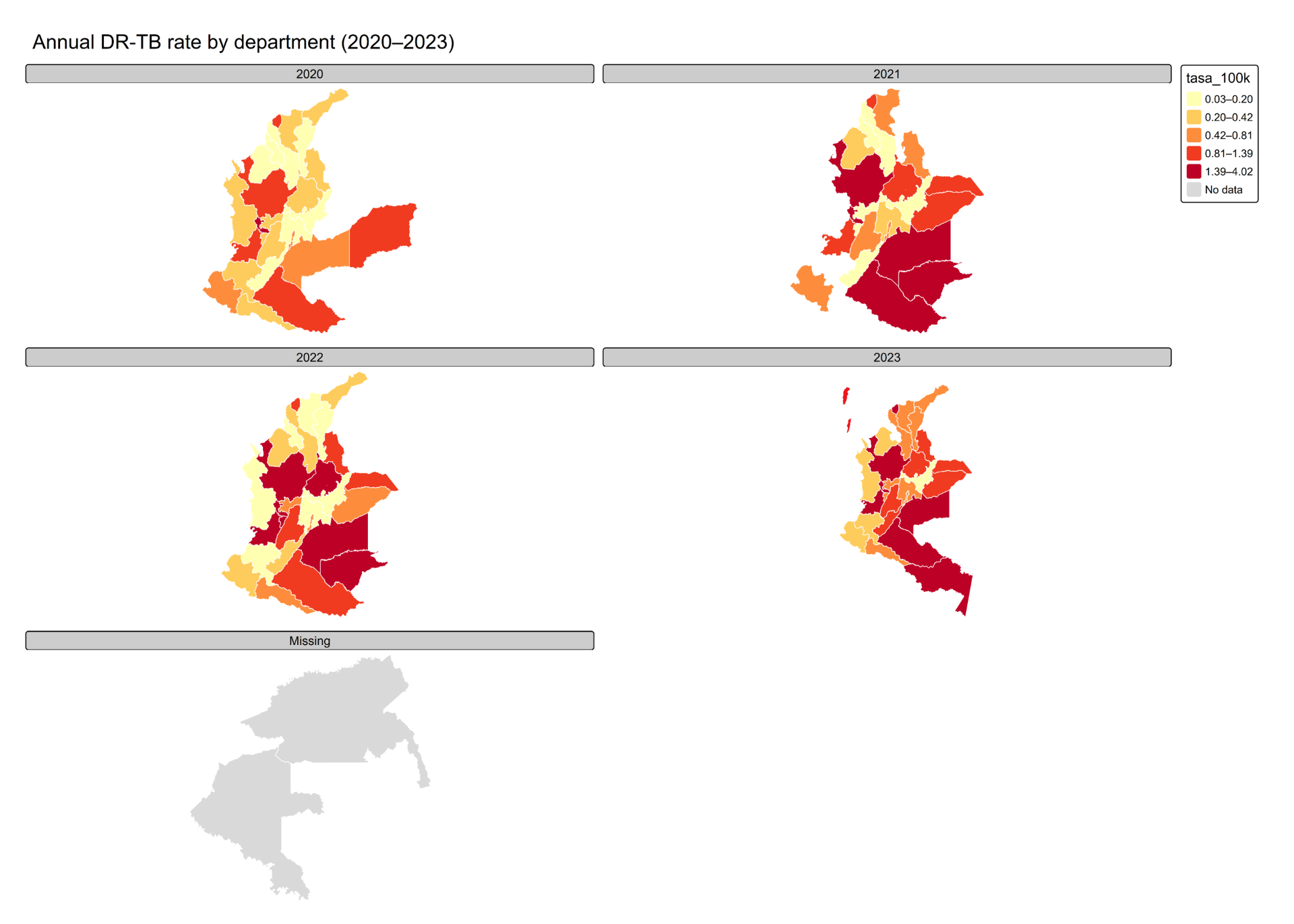

Supplement: Supplementary file 1 [file tropicalmed-10-00351-s001.zip › thumbnail_Figura S1 final.png]
